# Post-transplant diabetes mellitus requiring insulin identifies high-risk outcomes after solid organ transplantation

**DOI:** 10.1210/clinem/dgag217

**Published:** 2026-05-26

**Authors:** Rossana Caldara, Davide Catarinella, Chiara Gremizzi, Stefano Tentori, Vera Paloschi, Sarah Williford, Francesca Rusconi, Lorenzo Piemonti

**Affiliations:** Diabetes Research Institute and Clinic Unit of Regenerative Medicine and Organ Transplants, Istituto di Ricovero e Cura a Carattere Scientifico (IRCCS) Ospedale San Raffaele, Milan 20132, Italy; Diabetes Research Institute and Clinic Unit of Regenerative Medicine and Organ Transplants, Istituto di Ricovero e Cura a Carattere Scientifico (IRCCS) Ospedale San Raffaele, Milan 20132, Italy; Diabetes Research Institute and Clinic Unit of Regenerative Medicine and Organ Transplants, Istituto di Ricovero e Cura a Carattere Scientifico (IRCCS) Ospedale San Raffaele, Milan 20132, Italy; Diabetes Research Institute and Clinic Unit of Regenerative Medicine and Organ Transplants, Istituto di Ricovero e Cura a Carattere Scientifico (IRCCS) Ospedale San Raffaele, Milan 20132, Italy; Diabetes Research Institute and Clinic Unit of Regenerative Medicine and Organ Transplants, Istituto di Ricovero e Cura a Carattere Scientifico (IRCCS) Ospedale San Raffaele, Milan 20132, Italy; TriNetX LLC, Cambridge, MA 02140, USA; TriNetX Europe BV, 9830 Sint-Martens-Latem, Belgium; Diabetes Research Institute and Clinic Unit of Regenerative Medicine and Organ Transplants, Istituto di Ricovero e Cura a Carattere Scientifico (IRCCS) Ospedale San Raffaele, Milan 20132, Italy; Università Vita-Salute San Raffaele, Milan 20132, Italy

**Keywords:** post-transplant diabetes mellitus, solid organ transplantation, graft outcomes, insulin therapy, real-world data, propensity score matching

## Abstract

**Aims/Hypothesis:**

Post-transplant diabetes mellitus (PTDM) is a common complication after solid organ transplantation, but its long-term prognostic significance and the clinical implications of post-transplant antidiabetic treatment patterns remain incompletely understood. We aimed to evaluate PTDM incidence, determinants, treatment patterns, and associated long-term outcomes using large-scale real-world data.

**Methods:**

We analyzed adult solid organ transplant recipients without preexisting diabetes within the TriNetX Global Collaborative Network (2006-2024). PTDM incidence and risk factors were assessed using time-to-event analyses. Clinical outcomes were evaluated using propensity score–matched cohorts. Antidiabetic treatment patterns were characterized using treatment pathway analyses and outcomes were compared according to insulin vs non–insulin-based management among recipients with PTDM.

**Results:**

Among 263 325 recipients, PTDM incidence increased over time and varied markedly by transplanted organ, transplant era, and geographic region. Older age, male sex, adiposity, baseline dysglycemia, dyslipidemia, reduced renal function, and immunosuppressive exposure were independently associated with PTDM. After matching, PTDM was associated with higher long-term risks of mortality, graft failure, graft rejection, cardiovascular events, infections, malignancies, and renal–metabolic complications, whereas early post-transplant risks were largely comparable. Antidiabetic treatment patterns were heterogeneous and organ-specific. Insulin-treated PTDM recipients experienced substantially worse graft and patient outcomes than non–insulin-treated recipients, despite similar long-term glycemic control, indicating that insulin requirement identifies a high-risk metabolic phenotype. Findings were consistent in single organ transplant–specific sensitivity analyses.

**Conclusion/Interpretation:**

PTDM acts as a chronic modifier of long-term outcomes after solid organ transplantation. Insulin-treated PTDM identifies a subgroup at particularly high risk, underscoring the need for improved metabolic risk stratification and tailored management strategies in transplant recipients.

Post-transplant diabetes mellitus (PTDM) is a frequent metabolic complication after solid organ transplantation (1-3) and reflects the interplay between recipient vulnerability, peritransplant stressors, and the diabetogenic effects of immunosuppressive therapies, particularly calcineurin inhibitors and corticosteroids (4-10). Although PTDM has been associated with adverse outcomes, its true prognostic impact on graft and patient survival remains incompletely defined (11-14). Specifically, it is unclear whether PTDM primarily reflects baseline recipient frailty, contributes to early post-transplant complications, or instead represents a progressive modifier of long-term graft and patient risk (15-22). Prior studies have reported inconsistent findings, often limited by single-center design, organ-specific cohorts, heterogeneous definitions, short follow-up, and incomplete adjustment for baseline metabolic risk (13, 23-28). The limitation of prior organ-specific studies lies not in their organ-focused design per se, but in the resulting inability to benchmark PTDM incidence, risk determinants, and outcome associations directly across organ types within a unified analytical framework. Pooled analyses and organ-specific investigations serve complementary rather than competing scientific purposes: the former enable cross-organ comparisons with consistent methodology and adequate statistical power, whereas the latter allow mechanistic characterization of organ-specific pathophysiology in depth. At the same time, contemporary transplant recipients are older, metabolically more complex, and exposed to evolving immunosuppressive regimens designed to maximize graft survival. These shifts may influence both PTDM incidence and its clinical consequences. In addition, post-transplant glycemic management varies across centers and organ types (29, 30). Insulin therapy is frequently initiated early after PTDM diagnosis, yet whether insulin requirement identifies a subgroup with disproportionate long-term graft and patient risk—independent of achieved glycemic control—has not been systematically examined (31, 32). To address these gaps, we analyzed a large multinational cohort of adult solid organ transplant recipients without preexisting diabetes to characterize PTDM incidence and organ-specific heterogeneity, and to examine its short- and long-term associations with graft and patient outcomes. We additionally evaluated post-transplant antidiabetic treatment patterns, with particular focus on insulin use as a potential marker of high-risk metabolic phenotype.

## Materials and methods

### Data source and study design

This retrospective cohort study used data from the TriNetX Global Collaborative Network, a multinational federated platform aggregating de-identified electronic health records from academic medical centers and healthcare systems across North America, Europe, and other regions. The network provides harmonized longitudinal data on demographics, diagnoses, procedures, medications, laboratory results, and mortality (33). Analyses were performed within the secure TriNetX Analytics environment using de-identified data. Under U.S. Department of Health and Human Services guidance (45 CFR §46), analyses of de-identified data do not constitute human subjects research and therefore do not require institutional review board approval or informed consent. The study was conducted in accordance with STROBE guidelines. Detailed data provenance, cohort definitions, and analytic specifications are provided in the Supplementary Methods (34).

### Study Population

Adult (≥18 years) recipients of kidney, liver, heart, lung, combined heart–lung, or intestinal transplantation recorded between January 1, 2006, and December 31, 2024, were identified. Transplantation was ascertained using standardized ICD-10-CM diagnosis codes and CPT-4 procedural codes specific to each organ type, as detailed in Supplementary Methods Table S1 (34). Recipients were required to have at least 1 healthcare encounter within 12 months before transplantation. Individuals with evidence of preexisting diabetes prior to transplantation were excluded. Pretransplant diabetes exclusion was based primarily on the absence of diabetes-related ICD-10 diagnostic codes prior to transplantation. Although laboratory data and glucose-lowering medication records were reviewed descriptively when available, these variables were not used as mandatory exclusion criteria because of substantial heterogeneity in baseline laboratory capture across participating healthcare organizations. Cohort entry was defined as the date of transplantation. For PTDM analyses, a 90-day post-transplant landmark was applied. Recipients were followed until outcome occurrence, death, or last recorded activity. Combined heart–lung and intestinal recipients were included in pooled analyses but excluded from organ-specific models due to limited sample size.

### Definition of post-transplant diabetes Mellitus

PTDM was defined in accordance with the Third International PTDM Consensus (1) as incident diabetes occurring ≥90 days after transplantation. Diagnosis required either an ICD-10 code for diabetes mellitus (E08-E13) or laboratory criteria (HbA1c ≥ 6.5% or 2-hour plasma glucose ≥200 mg/dL on oral glucose tolerance testing). Consistent with consensus recommendations, glucose-lowering medications were not used as diagnostic criteria. Given the federated EHR-based nature of the TriNetX platform, formal OGTT data are not systematically available; the combined use of diagnostic codes and laboratory thresholds represents a prespecified operational adaptation of the consensus framework, consistent with prior large-scale real-world PTDM analyses. Individuals meeting criteria prior to transplantation or within the first 90 days post-transplant were not classified as having PTDM. In time-to-event models, PTDM was treated as a time-dependent exposure. The 90-day threshold was selected on the basis of both clinical and methodological considerations. Clinically, the majority of solid organ transplant recipients reach a degree of immunosuppressive stabilization—with calcineurin inhibitor levels titrated to target range and glucocorticoid tapers substantially completed—within 90 days of transplantation. Accordingly, the Third International PTDM Consensus explicitly endorses a ≥90-day diagnostic window, superseding the 45-day threshold proposed in the 2013 guidance, to ensure that PTDM is ascertained after resolution of the acute hyperglycemic milieu associated with induction immunosuppression and perioperative stress (1). Methodologically, the 90-day landmark also serves to mitigate immortal time bias: by restricting follow-up to individuals who survive and remain event-free through day 90, the analysis ensures that PTDM status and subsequent outcomes are not confounded by differential early post-transplant mortality.

### Baseline variables

Baseline variables were assessed within 12 months prior to transplantation and included demographics, body mass index, metabolic parameters, cardiometabolic comorbidities, laboratory biomarkers, and pretransplant medication exposures. For laboratory variables, the value closest to transplantation was used. Indicators of laboratory data availability were incorporated into propensity score models to account for heterogeneity in data capture. Covariates were selected a priori based on clinical relevance.

### Outcomes and analytical strategy

Outcomes included all-cause mortality, graft rejection, graft failure, and major post-transplant complications (cardiovascular events, severe infections, malignancies, and renal–metabolic deterioration). Outcomes were defined using prespecified ICD-10-CM diagnostic code clusters, supplemented where applicable by CPT/HCPCS procedural codes and LOINC laboratory concepts, as detailed in Supplementary Methods Table S4 (34). Initial comparisons were conducted in the overall cohort using absolute risks and time-to-event analyses. To reduce confounding, 1:1 propensity score matching was performed at the 90-day landmark using nearest-neighbor matching with caliper restriction. Covariate balance was assessed using standardized mean differences. Early (1-year) and long-term (5-year) risks were estimated overall and stratified by transplanted organ (kidney, liver, heart, lung). Post-transplant immunosuppressive exposures—including calcineurin inhibitors, mTOR inhibitors, and systemic corticosteroids—were incorporated into adjusted models as time-dependent covariates.

### Antidiabetic therapies and outcome analyses

Among recipients with PTDM, use of glucose-lowering therapies was identified based on documented prescriptions. Treatment sequences were reconstructed using the TriNetX Treatment Pathways framework. Determinants of drug initiation were evaluated using multivariable Cox models including demographic, metabolic, renal, cardiovascular, and transplant-related variables. Because insulin use may reflect greater metabolic severity, it was analyzed as a marker rather than a causal exposure. Recipients with PTDM were categorized according to insulin vs noninsulin management. Follow-up for treatment-based analyses began at PTDM diagnosis. Propensity score matching was used to improve comparability.

### Statistical analysis

All analyses were conducted within the TriNetX Analytics environment. Continuous variables are reported as mean ± standard deviation or median (interquartile range), as appropriate, and categorical variables as counts and percentages. Time-to-event outcomes were estimated using Kaplan–Meier methods and compared using Cox proportional hazards models. Time zero was defined according to the prespecified analytical framework: 90 days after transplantation for analyses evaluating PTDM incidence and PTDM-associated outcomes, and the date of PTDM diagnosis for treatment-based analyses. Hazard ratios (HRs) are reported with 95% confidence intervals (CIs). In propensity score–matched analyses, covariate balance was assessed using standardized mean differences (SMDs), with values <0.10 considered indicative of adequate balance. Continuous covariates were modelled using prespecified clinically meaningful increments. All statistical tests were two-sided, and a nominal *P* value <.05 was considered statistically significant. Assumptions of proportional hazards were evaluated within the TriNetX analytic framework. Additional details regarding model specifications, matching procedures, covariate handling, censoring rules, and sensitivity analyses are provided in the Supplementary Methods (34).

## Results

### Cohort description

Between 2006 and 2024, 263,325 adult solid organ transplant recipients without documented pretransplant diabetes were identified within the TriNetX Global Collaborative Network. Detailed baseline characteristics are presented in Table 1. Kidney transplantation was the most common procedure, followed by liver, heart, lung, combined heart–lung, and intestinal transplantation. Recipients with nonspecific transplant coding were included in pooled analyses only. The mean age at transplantation was 48.4 ± 18.9 years and 57.6% of recipients were male, with substantial variation across organ types. The cohort was predominantly white, although racial distribution differed by transplanted organ. Baseline glycemic indices were within the nondiabetic range by design. Body mass index and cardiometabolic comorbidities—including hypertension, dyslipidaemia, obesity, and chronic kidney disease—were common and showed organ-specific variation. Laboratory profiles reflected expected organ-related patterns, such as higher transaminases in liver recipients and reduced renal function in kidney recipients. Availability of laboratory data varied across variables, consistent with real-world electronic health record capture (Table S1) (35).

**Table 1:** Baseline characteristics of solid organ transplant recipients without pretransplant diabetes

Characteristic	All organs (*n* = 263 325)	Kidney (*n* = 139 477)	Liver (*n* = 56 106)	Heart (*n* = 17 949)	Lung (*n* = 11 322)	Heart–Lung (*n* = 793)	Intestine (*n* = 381)
**Age at transplant, years**	48.4 ± 18.9	48.6 ± 17.2	48.2 ± 20.5	44.7 ± 24.0	56.7 ± 14.9	46.6 ± 21.5	35.4 ± 23.9
**Current age, years**	56.6 ± 19.2	56.9 ± 17.5	56.3 ± 19.6	52.7 ± 24.5	64.2 ± 15.0	52.4 ± 21.5	43.6 ± 24.3
**Male sex, *n* (%)**	148 408 (57.6)	72 745 (56.0)	31 659 (59.0)	11 549 (66.7)	6033 (54.4)	493 (55.9)	149 (40.0)
**Race**							
**White, *n* (%)**	138 423 (53.8)	62 859 (46.7)	33 320 (63.0)	9760 (56.4)	7130 (64.3)	467 (52.0)	232 (64.0)
**Black/African American, *n* (%)**	37 976 (14.8)	23 752 (18.4)	3977 (7.1)	2683 (15.5)	1022 (9.2)	102 (11.0)	62 (17.0)
**Asian, *n* (%)**	9972 (3.9)	6360 (4.9)	1904 (3.9)	390 (2.3)	166 (1.5)	24 (3.0)	10 (3.0)
**HbA1c, %**	5.4 ± 1.0	5.45 ± 1.01	5.03 ± 0.99	5.81 ± 0.88	5.70 ± 0.78	5.70 ± 0.99	5.48 ± 0.39
**Fasting glucose, mg/dL**	109 ± 38	114 ± 42	121 ± 49	110 ± 40	110 ± 38	111 ± 41	97 ± 27
**Body weight, kg**	79.8 ± 21.1	79.4 ± 20.7	81.6 ± 21.8	83.0 ± 22.7	76.2 ± 18.1	77.1 ± 22.2	74.4 ± 24.7
**Body mass index, kg/m^2^**	27.3 ± 6.15	27.5 ± 6.04	27.4 ± 6.45	26.9 ± 6.51	26.4 ± 5.36	26.1 ± 5.9	25.1 ± 7.66
**Obesity, *n* (%)**	21 555 (9)	10 336 (8)	5157 (10)	1528 (9)	1282 (12)	147 (16)	35 (9)
**Hypertension, *n* (%)**	101 053 (41)	66 583 (52)	14 038 (26)	5963 (34)	3498 (32)	453 (51)	117 (31)
**Systolic BP, mmHg**	127 ± 22.5	135 ± 22.7	124 ± 20.7	112 ± 22.4	125 ± 18.9	122 ± 48.9	121 ± 18.9
**Diastolic BP, mmHg**	75.3 ± 14.2	78.5 ± 14.1	72.1 ± 13.8	70.9 ± 15.9	74.9 ± 11.7	77.4 ± 16.1	74.1 ± 13.9
**Heart rate, bpm**	81.9 ± 17.6	80.2 ± 15.8	82.9 ± 18.8	88.2 ± 21.5	86.2 ± 16.9	90.7 ± 17.8	87.6 ± 18.7
**Dyslipidaemia, *n* (%)**	43 192 (17)	26 193 (20)	5455 (10)	4090 (24)	2403 (22)	320 (36)	61 (16)
**Total cholesterol, mg/dL**	166 ± 59.6	174 ± 48.9	151 ± 76.9	152 ± 48.2	179 ± 46.2	160 ± 47.8	155 ± 48.3
**Triglycerides, mg/dL**	140 ± 106	156 ± 118	116 ± 95	125 ± 94	127 ± 80	140 ± 87	119 ± 84
**Chronic kidney disease, *n* (%)**	91 350 (37)	73 127 (57)	6484 (12)	3047 (18)	1070 (10)	264 (30)	43 (12)
**Serum creatinine, mg/dL**	3.12 ± 4.73	4.46 ± 5.04	1.27 ± 2.37	1.32 ± 3.52	1.10 ± 3.54	1.44 ± 3.66	1.64 ± 10.7
**eGFR (MDRD), mL/min/1.73 m^2^**	53.5 ± 40.2	34.3 ± 30.3	71.8 ± 35.7	67.0 ± 29.7	83.9 ± 33.7	66.2 ± 32.9	85.5 ± 34.0
**Ischemic heart disease, *n* (%)**	28 284 (11)	12 896 (10)	4692 (9)	4312 (25)	2239 (20)	293 (33)	34 (9)
**Viral hepatitis (B15–B19), n (%)**	15 309 (6)	2976 (2)	9512 (18)	235 (1)	201 (2)	20 (2)	10 (3)
**ALT, U/L**	53.2 ± 206	24.9 ± 58.2	154 ± 518	41.9 ± 149	26.9 ± 23.7	27.8 ± 28.6	41.1 ± 49.9
**AST, U/L**	63.8 ± 315	25.6 ± 75.1	216 ± 841	45.9 ± 186	28.0 ± 47.5	30.9 ± 48.6	39.5 ± 35.6

Baseline characteristics were assessed within the 365 days preceding transplantation. Values are reported as mean ± standard deviation (SD) or number (percentage), according to data availability within the baseline window. Obesity was defined based on diagnostic codes rather than BMI thresholds. Laboratory values correspond to the measurement closest to transplantation. Estimated glomerular filtration rate (eGFR) was calculated using the MDRD equation.

Abbreviations: BMI, body mass index; eGFR, estimated glomerular filtration rate; HbA1c, glycated haemoglobin; MDRD, Modification of Diet in Renal Disease.

### Incidence and risk of PTDM

Among 263 325 solid organ transplant recipients without pretransplant diabetes, the cumulative incidence of PTDM was 6.3% at 1 year, 19.9% at 5 years, and 31.5% at 10 years, with follow-up beginning at the predefined 90-day landmark (Table 2). These findings indicate that nearly 1 in 3 recipients developed PTDM within 10 years of transplantation. PTDM incidence differed substantially by transplanted organ (Table 2). Lung recipients demonstrated the highest cumulative incidence, followed by heart and combined heart–lung recipients, whereas liver recipients had consistently lower long-term incidence. Kidney transplant recipients showed intermediate risk. In Cox models adjusted for age, sex, and body mass index, these organ-specific differences persisted, confirming marked heterogeneity in PTDM risk across transplant types (Fig. 1; Table 2; Tables S2 and S3) (35). Across transplant eras, 5-year cumulative incidence decreased from earlier to more recent eras, whereas 1-year incidence showed smaller changes over time. Consistently, adjusted hazard ratios (aHRs) declined across successive eras compared with the 2006-2010 reference period (Fig. 1; Table 2). These patterns were most evident among liver and kidney transplant recipients, whereas lung transplant recipients continued to show higher cumulative incidence and elevated adjusted hazards across eras (Table S2) (35). Geographic differences were observed, with higher 5- and 10-year cumulative incidence in the United States network, intermediate values in the Asia–Pacific region, and lower incidence in EMEA and LATAM (Table 2). Region-specific Cox models showed corresponding variation in adjusted PTDM risk by organ type (Table S3) (35); however, estimates for several organ–region strata were imprecise because of smaller sample sizes and should be interpreted cautiously.

**Figure 1:**
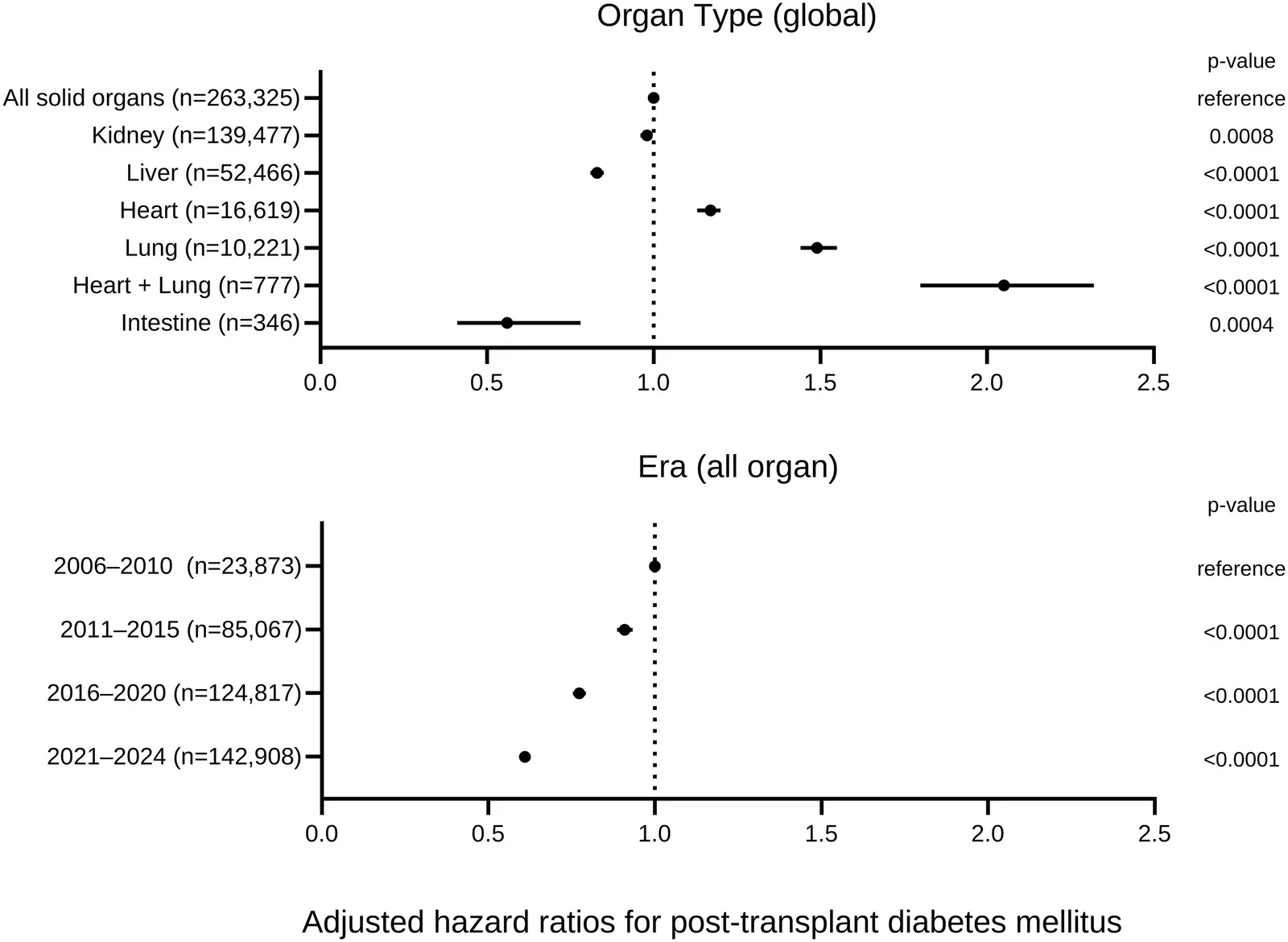
Adjusted hazard ratios (aHRs) for post-transplant diabetes mellitus (PTDM) by organ type and transplant era. Forest plots displaying aHRs for PTDM derived from multivariable Cox proportional hazards models. PTDM was defined as incident diabetes occurring ≥90 days after transplantation, based on the first documentation of an ICD-10 diabetes code (E08-E13) or qualifying laboratory criteria (HbA1c ≥ 6.5% or 2-hour plasma glucose ≥200 mg/dL on oral glucose tolerance testing). All models were adjusted for age at transplantation, sex, and baseline body mass index (BMI). The upper panel shows organ-specific aHRs, with the overall solid organ transplant cohort serving as the reference category (aHR = 1.00). The lower panel shows era-specific aHRs relative to the 2006–2010 transplant era (reference).

**Table 2:** Kaplan–Meier cumulative incidence estimates of PTDM from day 90 post-transplant, by organ type, transplant era, and geographic region

Category	Subgroup	*N*	1-year (%)	5-year (%)	10-year (%)	Median FU days (IQR)
Organ type	Kidney	139 477	5.7	19.7	32.2	1630 (2456)
	Liver	56 106	5.8	17.7	26.4	1398 (2445)
	Heart	21 949	7.1	19.9	30.9	1274 (2357)
	Lung	11 322	11.4	28.2	40.4	976 (1768)
	Heart–lung	793	7.1	19.8	33.1	1130 (1884)
	Intestine	381	4.2	11.4	15.2	1161 (2556)
	**All organs**	**263 325**	**6.3**	**19.9**	**31.5**	**1527 (2456)**
Era	2006-2010	28 900	5.5	20.2	32.0	4234 (3870)
	2011-2015	85 067	5.9	19.3	30.3	3150 (2610)
	2016-2020	130 441	5.2	17.9	30.2	2052 (1847)
	2021-2024	147 133	5.0	18.2		953 (1236)
Region (all organs)	United States	203 610	7.4	22.7	35.2	1644 (2504)
	EMEA	29 994	4.5	9.5	15.6	1248 (2253)
	LATAM	19 683	2.1	8.0	10.5	994 (1865)
	APAC	6685	5.4	20.3	32.1	1366 (2649)
Region (kidney only)	United States	106 399	6.6	22.1	35.7	1743 (2489)
	EMEA	14 644	2.5	10.3	17.7	1288 (2219)
	LATAM	11 109	2.2	9.1	11.6	1225 (1981)
	APAC	4515	5.2	20.6	33.1	1736 (2925)

PTDM was defined as the first incident occurrence occurring ≥90 days after transplantation, based on ICD-10 codes E08-E13 or qualifying laboratory criteria (HbA1c ≥ 6.5% or 2-hour oral glucose tolerance test ≥200 mg/dL). *N* denotes the number of individuals at risk at cohort entry (day 90 post-transplant). Cumulative incidence estimates were calculated as 1 minus Kaplan–Meier event-free survival and therefore differ from crude proportions due to right censoring. Ten-year cumulative incidence could not be estimated for the 2021-2024 era because of insufficient follow-up duration. Median follow-up reflects observed follow-up time and does not imply complete follow-up to the corresponding time horizon.

Abbreviations: BMI, body mass index; eGFR, estimated glomerular filtration rate; EMEA, Europe, the Middle East, and Africa; FU, follow-up; IQR, interquartile range; HbA1c, glycated haemoglobin; LATAM, Latin America; MDRD, Modification of Diet in Renal Disease; PTDM, post-transplant diabetes mellitus.

### Baseline factors associated with incident PTDM

Across the overall transplant cohort, multiple baseline characteristics were independently associated with incident PTDM (Table 3). Older age and male sex were consistent predictors; in fully adjusted models, PTDM risk increased by 21% per decade of age (aHR 1.21, 95% CI 1.20-1.22), and male recipients had a modestly higher risk than females (aHR 1.13, 95% CI 1.11-1.14). Markers of metabolic vulnerability demonstrated the strongest associations. Higher HbA1c and fasting glucose levels, impaired fasting glucose, impaired glucose tolerance, and prediabetes were all robust predictors, with prediabetes conferring nearly a 2-fold increased risk (aHR 1.97, 95% CI 1.86-2.08). Adiposity showed a graded dose–response relationship. Dyslipidemia—particularly low HDL cholesterol and hypertriglyceridemia—as well as vitamin D deficiency and metabolic-associated fatty liver disease were also associated with increased PTDM risk. Cardiometabolic comorbidities and markers of organ dysfunction—including hypertension, coronary artery disease, heart failure, peripheral artery disease, reduced renal function, hypalbuminaemia, and anemia—were consistently associated with incident PTDM. Behavioral and psychosocial factors, including substance-use disorders and smoking, further contributed to risk; alcohol-related disorders showed the strongest behavioral association (aHR 3.06, 95% CI 2.85-3.29). Pretransplant medication exposures reflecting underlying comorbidity burden, including systemic corticosteroids and other cardiometabolic therapies—were associated with higher PTDM risk. In addition, time-dependent exposure to calcineurin inhibitors, mTOR inhibitors, and corticosteroids after transplantation was associated with increased PTDM incidence, consistent with their known diabetogenic effects. In organ-specific analyses adjusted for age and sex, the magnitude of associations varied across transplant types (Fig. 2). Glycemic and lipid parameters exerted stronger effects in heart and lung recipients, whereas renal function showed greater influence in kidney and lung transplantation. Detailed organ-specific estimates are provided in Table S4 (35).

**Figure 2:**
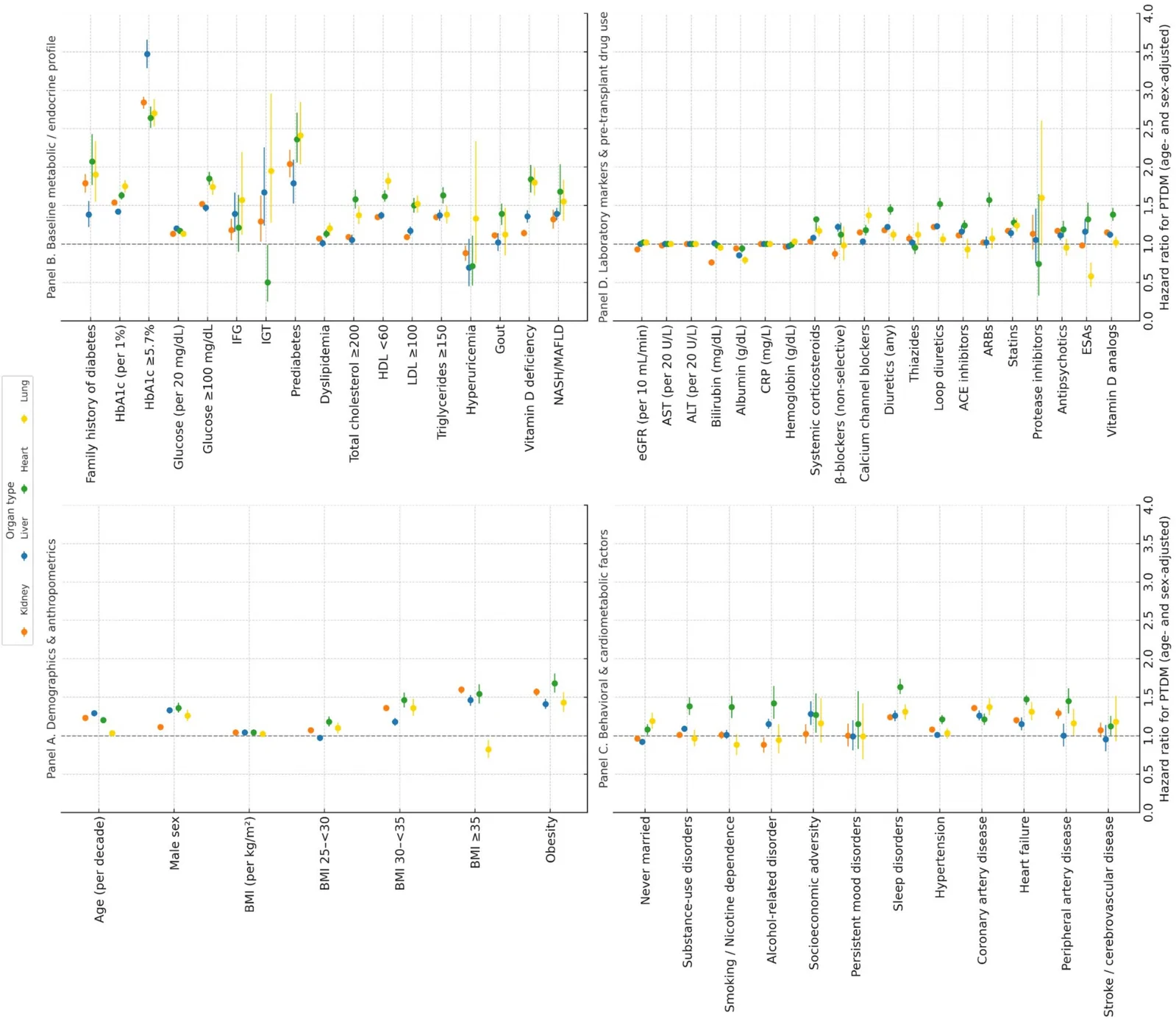
Factors associated with PTDM across organ types. Forest plots displaying organ-specific associations with PTDM derived from age- and sex-adjusted Cox proportional hazards models, fitted separately for kidney (*N* = 129 854), liver (*N* = 51 811), heart (*N* = 15 735), and lung (*N* = 9141) transplant recipients. The figure is organized into four panels: Panel A, demographic and anthropometric variables; Panel B, baseline metabolic and endocrine profile; Panel C, behavioral and cardiometabolic comorbidities; and Panel D, laboratory biomarkers and pretransplant medication exposures. For each factor, HRs with 95% confidence intervals are shown by organ type. All panels share a common *x*-axis scale (HR, 0-4) to facilitate direct comparison of effect sizes across organ groups. The vertical dashed line indicates HR = 1.0, representing no association with PTDM hazard. PTDM was defined as incident diabetes occurring ≥90 days after transplantation, based on ICD-10 diagnostic codes or laboratory criteria. Models were intentionally limited to adjustment for age and sex. The corresponding numerical estimates for all predictors are reported in Table S4 (35). *Abbreviations:* ACE, angiotensin-converting enzyme; ARB, angiotensin II receptor blocker; CI, confidence interval; CRP, C-reactive protein; eGFR, estimated glomerular filtration rate; ESAs, erythropoiesis-stimulating agents; HR, hazard ratio; IFG, impaired fasting glucose; IGT, impaired glucose tolerance; MAFLD, metabolic-associated fatty liver disease; NASH, non-alcoholic steatohepatitis; PTDM, post-transplant diabetes mellitus.

**Table 3:** Predictors of PTDM in all solid organ transplant recipients (*N* = 241 192)

Predictor	Unadjusted HR (95% CI)	*P*	Age- and sex–adjusted HR (95% CI)	*P*	Fully adjusted aHR*^a^* (95% CI)	*P*
**Demographics and Anthropometrics**						
**Age at transplant (per decade)**	1.23 (1.22-1.24)	<.0001	—	—	1.21 (1.20-1.22)	<.0001
**Male sex**	1.19 (1.18-1.21)	<.0001	—	—	1.13 (1.11-1.14)	<.0001
**BMI (per kg/m^2^)**	—	—	1.04 (1.04-1.04)	<.0001	1.04 (1.04-1.04)	<.0001
**BMI ≥ 25–<30**	—	—	1.09 (1.08-1.11)	<.0001	—	—
**BMI ≥ 30–<35**	—	—	1.32 (1.29-1.34)	<.0001	—	—
**BMI ≥ 35**	—	—	1.51 (1.48-1.54)	<.0001	—	—
**Obesity (ICD-10)**	—	—	1.54 (1.15-1.68)	<.0001	1.34 (1.31-1.37)	<.0001
**Baseline metabolic/endocrine profile**						
**Family history of diabetes**	—	—	1.70 (1.62-1.78)	<.0001	1.79 (1.70-1.87)	<.0001
**HbA1c (per 1%)**	—	—	1.52 (1.51-1.53)	<.0001	1.32 (1.31-1.34)	<.0001
**HbA1c ≥ 5.7%**	—	—	3.06 (3.00-3.11)	<.0001	—	—
**Glucose (per 20 mg/dL)**	—	—	1.13 (1.13-1.15)	<.0001	1.08 (1.08-1.10)	.0001
**Glucose ≥100 mg/dL**	—	—	1.58 (1.56-1.60)	<.0001	—	—
**IFG**	—	—	1.28 (1.18-1.40)	<.0001	1.19 (1.10-1.30)	<.0001
**IGT**	—	—	1.36 (1.17-1.58)	<.0001	1.29 (1.11-1.49)	.0008
**Prediabetes**	—	—	2.32 (2.20-2.45)	<.0001	1.97 (1.86-2.08)	<.0001
**Dyslipidaemia**	—	—	1.14 (1.12-1.15)	<.0001	1.10 (1.09-1.12)	<.0001
**Total cholesterol ≥ 200 mg/dL**	—	—	1.20 (1.18-1.23)	<.0001	1.17 (1.15-1.20)	<.0001
**HDL < 60 mg/dL**	—	—	1.50 (1.48-1.52)	<.0001	1.44 (1.42-1.46)	<.0001
**LDL ≥ 100 mg/dL**	—	—	1.24 (1.22-1.27)	<.0001	1.20 (1.17-1.22)	<.0001
**Triglycerides ≥ 150 mg/dL**	—	—	1.49 (1.46-1.52)	<.0001	1.37 (1.35-1.40)	<.0001
**Hyperuricemia**	—	—	1.04 (0.95-1.14)	.41	0.97 (0.89-1.07)	.56
**Gout**	—	—	1.16 (1.13-1.20)	<.0001	1.15 (1.11-1.18)	<.0001
**Vitamin D deficiency**	—	—	1.35 (1.32-1.39)	<.0001	1.37 (1.34-1.41)	<.0001
**NASH/MAFLD**	—	—	1.40 (1.35-1.46)	<.0001	1.44 (1.38-1.49)	<.0001
**Behavioral and psychosocial factors**						
**Never married**	—	—	0.97 (0.95-0.98)	<.0001	0.99 (0.98-1.01)	.49
**Substance-use disorders**	—	—	1.08 (1.05-1.10)	<.0001	1.17 (1.14-1.19)	<.0001
**Smoking**	—	—	1.03 (0.99-1.06)	0.12	1.11 (1.07-1.14)	<.0001
**Alcohol-related disorder**	—	—	1.12 (1.09-1.15)	<.0001	3.06 (2.85-3.29)	<.0001
**Socioeconomic adversity**	—	—	1.28 (1.20-1.37)	<.0001	1.34 (1.25-1.43)	<.0001
**Persistent mood disorders**	—	—	1.01 (0.92-1.11)	.88	1.03 (0.94-1.12)	.60
**Sleep disorders**	—	—	1.38 (1.35-1.41)	<.0001	1.58 (1.50-1.68)	<.0001
**Cardiometabolic comorbidities**						
**Hypertension**	—	—	1.05 (1.03-1.06)	<.0001	1.11 (1.10-1.13)	<.0001
**Coronary artery disease**	—	—	1.39 (1.36-1.42)	<.0001	1.35 (1.33-1.38)	<.0001
**Heart failure**	—	—	1.43 (1.41-1.46)	<.0001	1.35 (1.32-1.38)	<.0001
**Peripheral artery disease**	—	—	1.33 (1.28-1.38)	<.0001	1.34 (1.28-1.39)	<.0001
**Stroke**	—	—	1.15 (1.09-1.22)	<.0001	1.10 (1.04-1.16)	.001
**Laboratory/functional biomarkers**						
**eGFR (per 10 mL/min)**	—	—	0.97 (0.97-0.97)	<.0001	0.97 (0.96-0.97)	<.0001
**AST (per 20 U/L)**	—	—	1.00 (0.99-1.01)	.70	—	—
**ALT (per 20 U/L)**	—	—	1.00 (1.00-1.00)	<.0001	—	—
**Bilirubin**	—	—	1.00 (1.00-1.00)	.07	1.01 (1.00-1.01)	<.0001
**Albumin**	—	—	0.92 (0.91-0.93)	<.0001	0.90 (0.89-0.91)	<.0001
**CRP**	—	—	1.00 (1.00-1.00)	.01	1.00 (1.00-1.00)	.03
**Haemoglobin**	—	—	0.98 (0.98-0.98)	<.0001	0.96 (0.96-0.97)	<.0001
**Pretransplant drug use**						
**Systemic corticosteroids**	—	—	1.09 (1.07-1.10)	<.0001	1.12 (1.10-1.13)	<.0001
**β-blockers (non-selective)**	—	—	1.04 (1.00-1.07)	.03	1.16 (1.13-1.20)	<.0001
**Calcium channel blockers**	—	—	1.14 (1.13-1.16)	<.0001	1.15 (1.14-1.17)	<.0001
**Diuretics**	—	—	1.23 (1.22-1.25)	<.0001	1.25 (1.23-1.26)	<.0001
**Thiazides**	—	—	1.04 (1.01-1.08)	.01	1.02 (0.98-1.05)	.33
**Loop diuretics**	—	—	1.27 (1.25-1.28)	<.0001	1.28 (1.26-1.30)	<.0001
**ACE inhibitors**	—	—	1.16 (1.14-1.19)	<.0001	1.15 (1.12-1.17)	<.0001
**ARBs**	—	—	1.15 (1.13-1.18)	<.0001	1.11 (1.08-1.14)	<.0001
**Statins**	—	—	1.26 (1.24-1.29)	<.0001	1.17 (1.15-1.19)	<.0001
**Protease inhibitors**	—	—	1.19 (1.04-1.37)	.01	1.23 (1.06-1.42)	.005
**Antipsychotics**	—	—	1.07 (1.04-1.09)	<.0001	1.13 (1.10-1.15)	<.0001
**ESAs**	—	—	1.08 (1.04-1.11)	<.0001	1.13 (1.09-1.16)	<.0001
**Vitamin D analogues**	—	—	1.21 (1.19-1.23)	<.0001	1.20 (1.18-1.22)	<.0001
**Posttransplant drug use (time-dependent)**						
**Calcineurin inhibitors (0-365 days)**	—	—	—	—	1.78 (1.75-1.81)	<.0001
**mTOR inhibitors (30-365 days)**	—	—	—	—	1.47 (1.41-1.54)	<.0001
**Corticosteroids (0-90 days)**	—	—	—	—	1.44 (1.42-1.47)	<.0001
**Corticosteroids (90-365 days)**	—	—	—	—	1.87 (1.84-1.91)	<.0001

^*a*^Fully adjusted hazard ratios (aHRs) were estimated using Cox proportional hazards models including age, sex, body mass index (BMI), baseline glycated haemoglobin (HbA1c), fasting plasma glucose, and transplant organ type. Age and sex were first examined in univariable models and subsequently retained in all multivariable analyses. All remaining baseline covariates were initially evaluated in age- and sex-adjusted models and then re-estimated in the fully adjusted model. Post-transplant immunosuppressive exposures (calcineurin inhibitors, mTOR inhibitors, and systemic corticosteroids) were modelled as time-dependent covariates. PTDM was defined as the first occurrence of ICD-10 codes E08–E13 or a qualifying laboratory criterion (HbA1c ≥ 6.5% or 2-hour oral glucose tolerance test ≥200 mg/dL) occurring ≥90 days after transplantation. Baseline laboratory values were defined as the measurement closest to transplantation within the preceding 365 days.

Abbreviations: ACE, angiotensin-converting enzyme; ARBs, angiotensin II receptor blockers; BMI, body mass index; CRP, c-reactive protein; eGFR, estimated glomerular filtration rate; EMEA, Europe, the Middle East, and Africa; ESAs, erythropoiesis-stimulating agents; FU, follow-up; HbA1c, glycated haemoglobin; HDL, high-density lipoproteins; IFG, impaired fasting glucose; IGT, impaired glucose tolerance; IQR, interquartile range; LATAM, Latin America; MAFLD, metabolic-associated fatty liver disease; NASH, non-alcoholic steatohepatitis; PTDM, post-transplant diabetes mellitus.

### Primary graft and patient outcomes

In propensity score–matched analyses, PTDM was not associated with materially higher risk of early adverse outcomes. At 1-year, absolute risks of all-cause mortality, graft rejection, and graft failure were comparable between recipients with and without PTDM, with minimal absolute differences and neutral relative effect estimates (Table 4; Table S5) (35). In contrast, a clear divergence emerged over longer follow-up. By 5 years, PTDM was consistently associated with higher risks of all-cause mortality, graft rejection, and graft failure across both absolute-risk and time-to-event analyses (Table 4; Tables S6 and S7) (35). The composite endpoint of death or graft failure followed the same temporal pattern, with negligible differences at 1 year and a clinically meaningful excess risk at 5 years. Organ-specific analyses revealed heterogeneity in effect magnitude. Long-term associations were most pronounced among kidney and lung transplant recipients, more modest in heart transplantation, and largely neutral for graft rejection in liver recipients (Table S7) (35). Kaplan–Meier curves demonstrated progressive separation beyond the first post-transplant year, supporting a time-dependent pattern of risk (Fig. 3).

**Figure 3:**
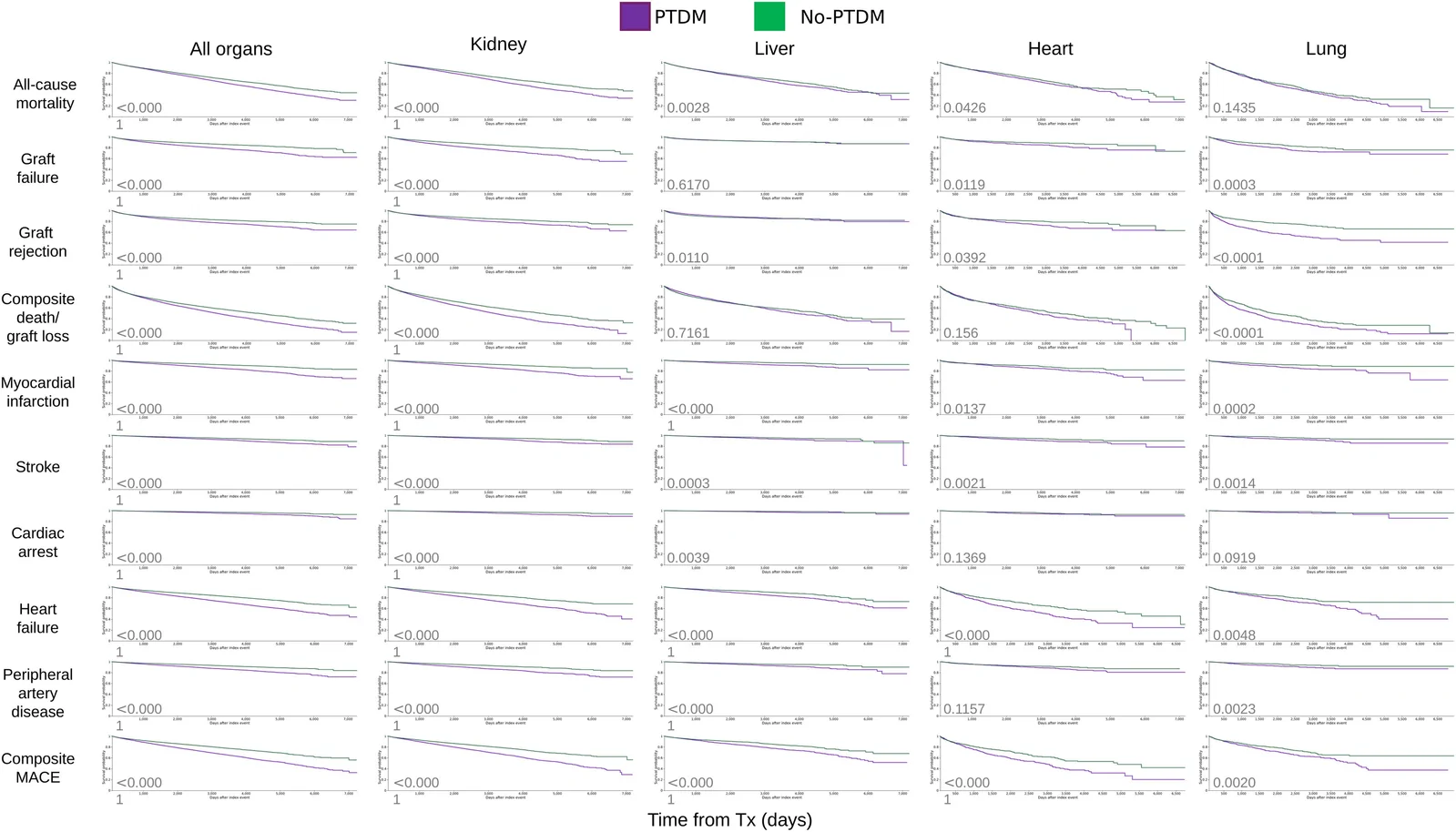
Clinical and cardiovascular outcomes associated with post-transplant diabetes mellitus (PTDM). Kaplan–Meier curves comparing solid organ transplant recipients with PTDM versus those without PTDM for all-cause mortality, graft failure, graft rejection, the composite endpoint of death or graft failure, and major cardiovascular events (myocardial infarction, stroke, cardiac arrest, heart failure, peripheral artery disease, and composite MACE). Analyses are shown for the overall transplant population and stratified by transplanted organ (kidney, liver, heart, and lung). Time is calculated from transplantation; between-group comparisons were performed using the log-rank test. Detailed outcome definitions and coding algorithms are reported in Supplementary Methods (34).

**Table 4:** Primary graft and patient outcomes in the propensity score–matched cohort: one-year and five-year absolute risks and adjusted hazard ratios associated with PTDM

Outcome	1-year AR (%) PTDM/no PTDM	RD % (95% CI)	RR (95% CI)	5-year AR (%) PTDM/no PTDM	RD % (95% CI)	RR (95% CI)	HR (95% CI)	*P*
All-cause mortality	3.82/3.61	0.21 (–0.05 to 0.47)	1.06 (0.99-1.13)	15.02/12.40	2.63 (2.16-3.10)	1.21 (1.17-1.25)	1.22 (1.18-1.26)	<.0001
Graft rejection	4.53/4.63	–0.10 (–0.41 to 0.21)	0.98 (0.91-1.05)	11.75/9.42	2.33 (1.87-2.79)	1.25 (1.19-1.30)	1.25 (1.20-1.31)	<.0001
Graft failure	3.30/2.91	0.39 (0.14-0.65)	1.14 (1.05-1.23)	9.89/6.67	3.22 (2.82-3.63)	1.48 (1.41-1.56)	1.50 (1.44-1.58)	<.0001
Composite: death or graft failure or graft rejection	8.28/8.41	–0.13 (–0.56 to 0.30)	0.99 (0.94-1.04)	25.35/21.13	4.22 (3.56-4.88)	1.20 (1.17-1.23)	1.22 (1.18-1.25)	<.0001

AR represent the cumulative incidence at 1 and 5 years in the propensity score–matched cohort. RD are expressed as percentage points with 95% CI, and RR are reported with 95% CI. HR were estimated using Cox proportional hazards models and reflect time-to-event risk over the follow-up period. All estimates are derived from propensity score–matched analyses comparing solid organ transplant recipients with PTDM vs those without PTDM. Organ-specific hazard ratios for kidney, liver, heart, and lung transplantation are reported in Table S7 (35). Statistical inference is based on both two-sided *P* values and 95% confidence intervals, with statistical significance inferred when the CI does not cross the null value.

Abbreviations: AR, absolute risk; CI, confidence interval; HR, hazard ratio; PTDM, post-transplant diabetes mellitus; RD, risk difference; RR, risk ratio.

### Cardiovascular, infectious, neoplastic, and renal–metabolic complications

Beyond graft-related outcomes, PTDM was associated with a broader burden of long-term complications across multiple clinical domains (Fig. 3; Table S6) (35). In propensity score–matched analyses, 5-year cardiovascular risk was consistently higher among recipients with PTDM, including major adverse cardiovascular events and their individual components. Infectious complications were likewise more frequent, particularly severe bacterial and fungal infections, including transplant-organ and urinary tract infections. In contrast, viral opportunistic infections were less common after matching. Associations with neoplastic outcomes were more modest but directionally consistent, with higher incidences of overall malignancy, nonmelanoma skin cancer, and post-transplant lymphoproliferative disorders. Hepatocellular carcinoma occurred less frequently among recipients with PTDM. Renal and metabolic complications demonstrated the most pronounced differences. PTDM was associated with increased risks of chronic kidney disease progression, transition to dialysis or end-stage renal disease, and acute metabolic events, including hyperglycemic crises, hypoglycemia, and electrolyte disturbances. Organ-specific sensitivity analyses showed broadly consistent directions of association across kidney, liver, heart, and lung transplantation (Fig. 3; Tables S7 (35); Figs. S1 and S2 (36)).

### Antidiabetic treatment patterns and determinants of drug class initiation

Among recipients who developed PTDM and received glucose-lowering therapy (n = 39 595), treatment patterns demonstrated substantial heterogeneity but followed a structured temporal progression (Fig. 4). Insulin was most initiated early after diagnosis, whereas use of noninsulin agents—including biguanides, DPP-4 inhibitors, GLP-1 receptor agonists, and SGLT2 inhibitors—increased over subsequent treatment lines. Combination therapy became progressively more frequent, consistent with stepwise intensification rather than isolated class switching. Multivariable models identified distinct, phenotype-driven determinants of drug class initiation (Fig. 4C; Table S8) (35). Higher glycated hemoglobin was consistently associated with greater likelihood of treatment across all classes. Preserved renal function favored initiation of non-insulin agents, whereas reduced estimated glomerular filtration rate and chronic kidney disease were associated with greater insulin use. Treatment patterns also varied by transplanted organ, with lung and liver recipients more frequently treated with insulin and heart recipients more likely to receive GLP-1 receptor agonists or SGLT2 inhibitors. These associations were directionally consistent across organ-specific sensitivity analyses (Supplementary Figs. S3-S6) (36). In outcome-oriented analyses, insulin-treated PTDM identified a subgroup at higher risk of adverse graft and patient outcomes. In propensity score–matched comparisons, insulin-treated recipients had higher 1-year and 5-year risks of mortality, graft rejection, graft failure, and the composite endpoint (Table 5; Fig. 5). Notably, longitudinal glycemic control converged between insulin-treated and non–insulin-treated recipients beyond the early postdiagnosis phase, supporting interpretation of insulin requirement as a marker of underlying metabolic severity rather than sustained differences in glycaemic exposure.

**Figure 4:**
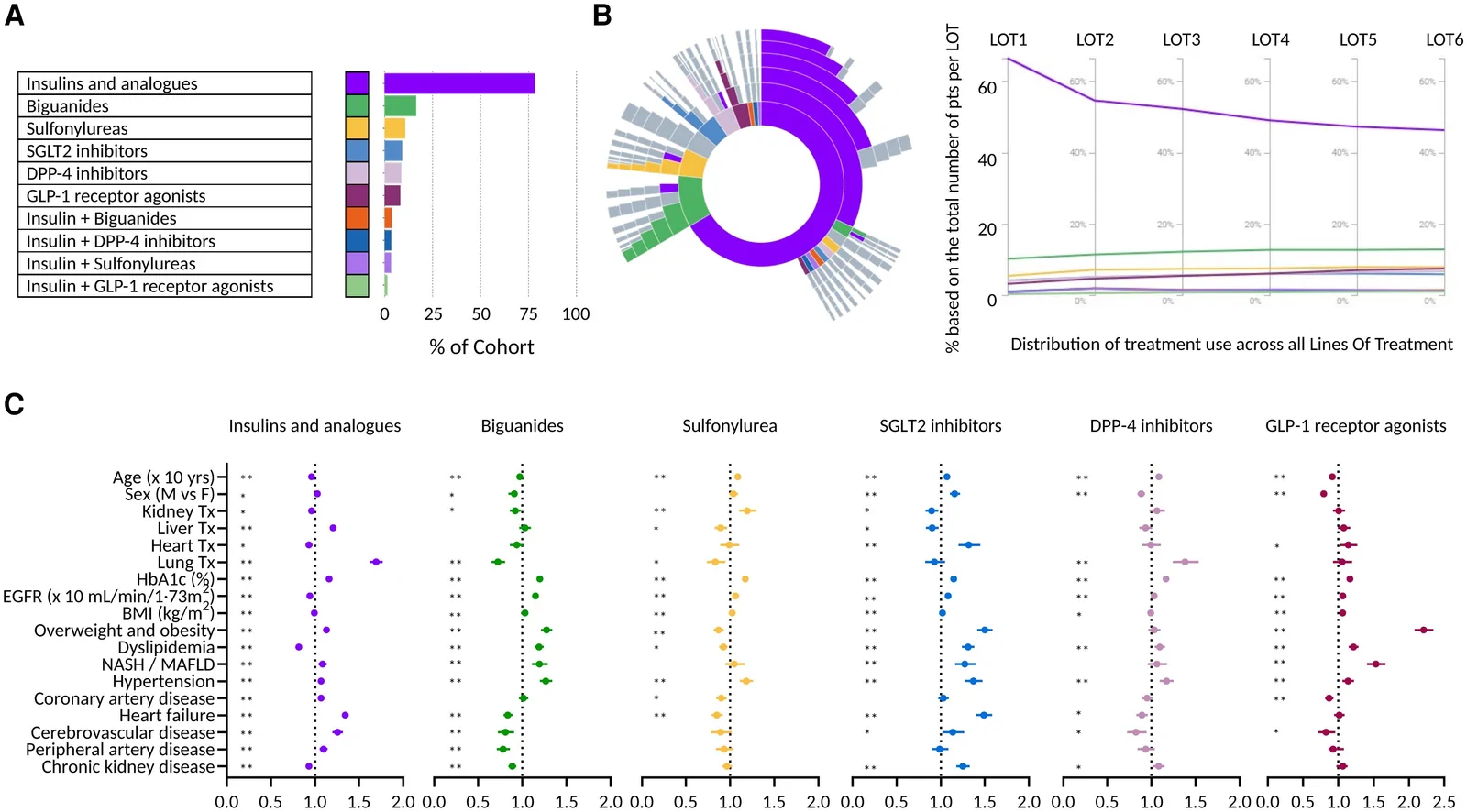
Antidiabetic treatment patterns and determinants of antidiabetic drug class use among solid organ transplant recipients with post-transplant diabetes mellitus (PTDM). *Panel A.* Distribution of antidiabetic drug classes with documented use after PTDM diagnosis in the overall solid organ transplant cohort (*n* = 39 595). Drug classes include insulins and analogues, biguanides, sulfonylureas, SGLT2 inhibitors, DPP-4 inhibitors, GLP-1 receptor agonists, and selected combination therapies. *Panel B.* Treatment pathways and temporal evolution of antidiabetic therapy following PTDM diagnosis. The circular diagram illustrates transitions between antidiabetic drug classes, with segment size proportional to the number of patients with documented use of each therapy and connecting arcs representing treatment changes over time. The accompanying line plot shows the proportion of patients receiving each drug class across successive lines of therapy (LOT1 to LOT6). Treatment pathway analyses were available for *n* = 24 291 patients with a mean follow-up of 1801 days (SD 1469) and a median follow-up of 1458 days (interquartile range 2125 days). Only pathways observed in ≥1% of patients are displayed. *Panel C.* Forest plots showing adjusted hazard ratios (aHRs) and 95% confidence intervals (CIs) for factors associated with documented use of each antidiabetic drug class. Estimates were derived from multivariable Cox proportional hazards models in the overall PTDM population (*n* = 39 197). Covariates included age, sex, transplanted organ type, BMI, renal function, glycaemic control, and major metabolic, cardiovascular, renal, and transplant-related comorbidities. HRs >1 indicate a higher likelihood of documented use of the corresponding drug class. * *P* < .05; ** *P* < .001.

**Figure 5:**
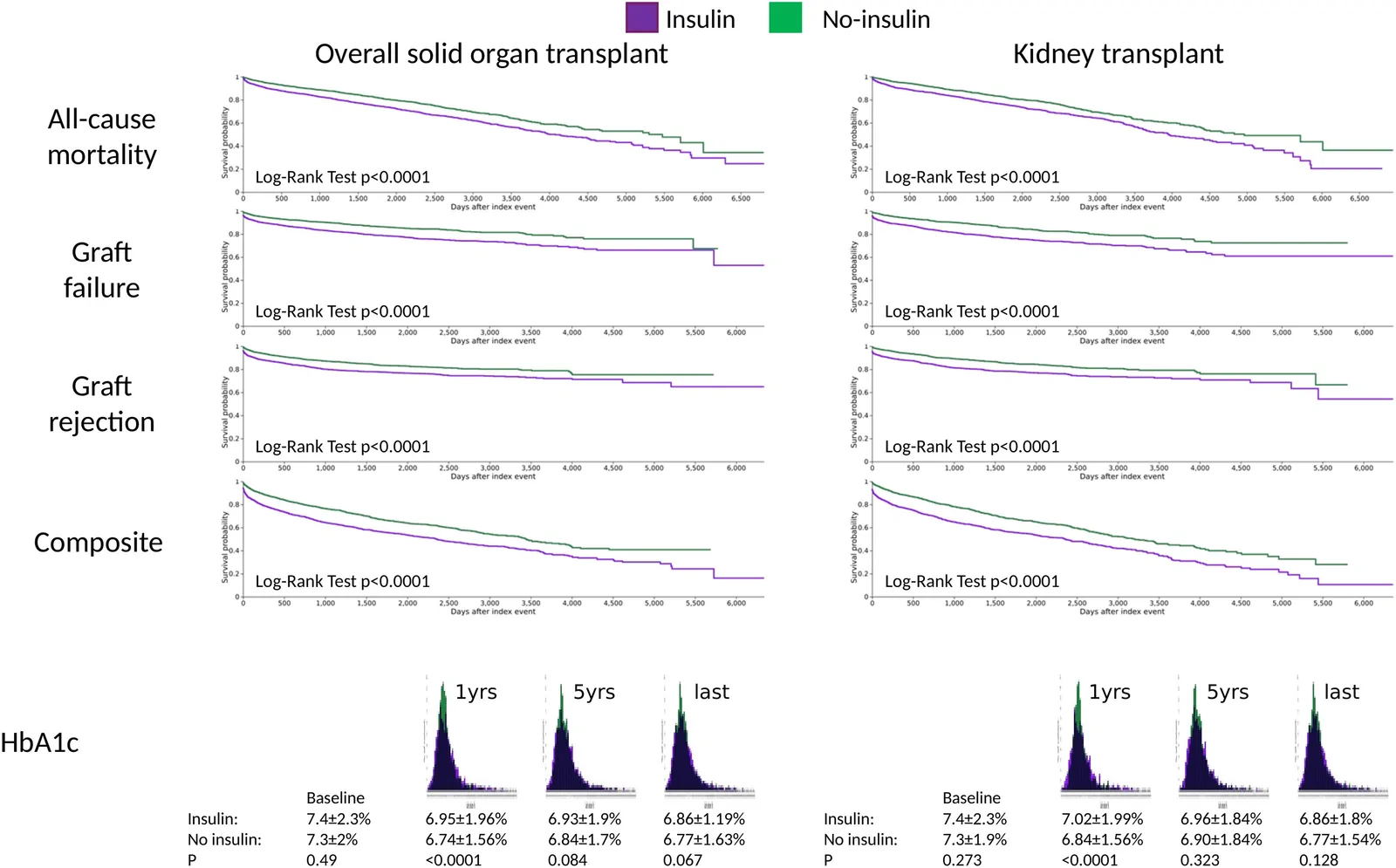
Clinical outcomes and longitudinal glycemic control according to insulin treatment among recipients with post-transplant diabetes mellitus (PTDM). Kaplan–Meier curves comparing solid organ transplant recipients with PTDM treated with insulin versus those not treated with insulin for all-cause mortality, graft failure, graft rejection, and the composite endpoint of death, graft failure, or graft rejection, shown for the overall transplant population and for kidney transplant recipients. Time is calculated from PTDM diagnosis; between-group comparisons were performed using the log-rank test. Lower panels show longitudinal distributions of glycated hemoglobin (HbA1c) at PTDM diagnosis, at 1 year, 5 years, and at last available follow-up, stratified by insulin treatment status. Between-group comparisons of HbA1c values were performed using two-sample *t* tests. The propensity score–matched PTDM cohort included 3261 insulin-treated and 3261 non–insulin-treated recipients in the overall population, and 2330 insulin-treated and 2330 non–insulin-treated recipients in the kidney transplant subgroup. Details of the propensity score matching procedure and covariate balance are provided in the Supplementary Methods (34).

**Table 5:** Primary graft and patient outcomes at 1 and 5 years in the propensity score–matched cohort, by insulin treatment in PTDM

Outcome	1-year AR (%) insulin/no insulin	RD % (95% CI)	RR (95% CI)	5-year AR (%) insulin/no insulin	RD % (95% CI)	RR (95% CI)	HR (95% CI)	*P*
All-cause mortality	10.01/5.34	4.66 (3.35-5.98)	1.87 (1.57-2.24)	20.06/13.61	6.44 (4.6-8.3)	1.47 (1.32-1.65)	1.43 (1.29-1.58)	.0031
Graft rejection	12.32/7.21	5.1 (3.43-6.77)	1.71 (1.43-2.04)	18.25/12.56	5.69 (3.66-7.71)	1.45 (1.27-1.66)	1.52 (1.32-1.76)	<.0001
Graft failure	10.49/5.64	4.84 (3.35-6.33)	1.86 (1.53-2.26)	16.67/10.22	6.45 (4.58-8.31)	1.63 (1.41-1.88)	1.68 (1.46-1.95)	.0026
Composite: death or graft failure or graft rejection	22.41/12.56	9.85 (7.65-12.1)	1.78 (1.56-2.04)	36.64/25.66	10.97 (8.30-13.68)	1.43 (1.31-1.56)	1.52 (1.38-1.68)	<.0001

Absolute risks (AR) represent the cumulative incidence at 1 and 5 years in the propensity score–matched cohort. Risk differences (RD) are expressed as percentage points with 95% confidence intervals (CI), and risk ratios (RR) are reported with 95% CI. Hazard ratios (HR) were estimated using Cox proportional hazards models and reflect time-to-event risk over the entire follow-up period. All estimates are derived from propensity score–matched analyses comparing transplant recipients with PTDM treated with insulin vs those not treated with insulin. Two-sided *P* values are reported. Statistical inference is based on both *P* values and 95% confidence intervals, with statistical significance inferred when the CI does not cross the null value.

Abbreviations: AR, absolute risk; CI, confidence interval; HR, hazard ratio; PTDM, post-transplant diabetes mellitus; RD, risk difference; RR, risk ratio.

## Discussion

In this large, real-world analysis of solid organ transplant recipients without preexisting diabetes, we provide a comprehensive characterization of PTDM and clarify its long-term clinical implications across organ types, eras, and geographic regions (37). Three principal findings emerge. First, PTDM is common and clinically heterogeneous, with substantial variation in incidence and risk across transplanted organs, transplant eras, and regions. Second, PTDM is not associated with worse early post-transplant outcomes but is consistently linked to a higher burden of adverse graft and patient outcomes over longer follow-up, supporting its role as a time-dependent modifier of long-term risk rather than an early driver of post-transplant events (15, 22, 27). Third, among recipients with PTDM, insulin-treated individuals represent a distinct high-risk metabolic phenotype characterized by worse outcomes despite convergence of glycemic control over time. Our findings refine the conceptual framework of PTDM in transplant medicine. After rigorous propensity score matching, PTDM was not associated with excess mortality, graft failure, or rejection at 1 year but was consistently associated with higher risks at 5 years and beyond. The delayed separation of outcome curves suggests that PTDM primarily amplifies cumulative metabolic and vascular injury rather than precipitating early post-transplant complications. This interpretation is biologically plausible: chronic hyperglycemia, insulin resistance, and related cardiometabolic disturbances exert progressive effects on endothelial function, immune competence, infection susceptibility, malignancy risk, and long-term graft integrity (38). Importantly, this time-dependent pattern may help reconcile inconsistencies in prior reports. Studies with shorter follow-up durations, organ-restricted cohorts, or limited statistical power may have underestimated the long-term impact of PTDM, whereas analyses incorporating extended follow-up and robust adjustment strategies reveal a clearer signal of cumulative risk. Framing PTDM as a chronic modifier of post-transplant trajectory, rather than as an acute complication, has practical implications for surveillance intensity, cardiovascular prevention, and long-term graft management.

By leveraging long-term follow-up in a large, multinational cohort, our analysis demonstrates that the clinical relevance of PTDM unfolds predominantly beyond the early post-transplant period. Rather than acting as an acute complication of transplantation, PTDM appears to reshape the long-term risk trajectory of recipients, with adverse effects emerging progressively over time. This temporal dimension reframes PTDM as a chronic modifier of transplant outcomes, with implications that extend well beyond initial graft stabilization.

The marked heterogeneity observed across transplanted organs underscores the complementary value of both pooled and organ-specific approaches to PTDM research. The strength of the present analysis lies in its capacity to benchmark incidence, risk determinants, and outcome associations directly across organ types within a consistent methodological framework—a comparison not achievable through aggregation of independent single-organ studies with heterogeneous designs. At the same time, our findings reinforce the scientific importance of dedicated organ-specific investigations to elucidate the biological mechanisms underlying these differences, and our organ-stratified sensitivity analyses were designed with this complementarity in mind. Differences in maintenance immunosuppressive regimens across transplanted organs, particularly regarding corticosteroid exposure and calcineurin inhibitor use, likely contribute to the observed heterogeneity in PTDM incidence and outcomes across organ groups. Importantly, post-transplant immunosuppressive exposures were incorporated into adjusted analyses as time-dependent covariates, supporting interpretation of the observed organ-specific differences beyond simple variation in immunosuppressive intensity alone. Marked heterogeneity was observed across transplanted organs. Lung and heart transplant recipients exhibited the highest PTDM incidence, whereas liver transplant recipients showed lower long-term incidence and more attenuated associations with selected outcomes. These differences likely reflect a complex interplay of baseline cardiometabolic vulnerability, intensity and composition of immunosuppressive regimens, competing risks, and organ-specific pathophysiology. For example, lung and heart recipients often carry a high pre-transplant cardiovascular burden and are exposed to sustained immunosuppressive intensity, whereas liver transplantation may partially modify metabolic milieu in selected recipients. Variations in surveillance intensity and post-transplant management strategies may further contribute to the observed patterns. Geographic heterogeneity reinforces the concept that PTDM risk is not solely biologically determined but also shaped by population characteristics, healthcare system structure, and local transplant practices. Differences in recipient selection, access to metabolic care, and immunosuppressive protocols likely contribute to the variation observed across regions. Importantly, the decline in PTDM risk across successive transplant eras—most evident among kidney and liver recipients—suggests that contemporary improvements in perioperative management, immunosuppressive tailoring, and risk stratification may have mitigated, but not eliminated, the metabolic burden of transplantation. Despite these improvements, PTDM remains common and prognostically relevant in modern cohorts. The persistence of long-term risk underscores that advances in graft survival have not been paralleled by equivalent progress in metabolic prevention. Collectively, these findings argue for sustained and structured metabolic surveillance throughout the transplant lifespan, with integration of cardiometabolic risk management into routine long-term transplant care rather than confinement to the early post-transplant phase (1).

A particularly novel and clinically relevant aspect of our study is the outcome-oriented evaluation of antidiabetic treatment patterns after PTDM diagnosis. Insulin therapy, frequently initiated early after disease onset, was consistently associated with worse graft and patient outcomes compared with non–insulin-based strategies. Crucially, however, longitudinal analyses demonstrated convergence of glycemic indices over time between insulin-treated and non–insulin-treated recipients. This finding argues against sustained differences in achieved glycemic control as the primary explanation for the observed excess risk. Instead, insulin requirement identifies a subgroup with greater underlying metabolic fragility. These recipients were characterized by features consistent with more advanced metabolic dysfunction, including reduced β-cell reserve, impaired renal function, and a higher burden of cardiometabolic comorbidities. Within this framework, insulin use should not be interpreted as a causal driver of adverse outcomes, but rather as a pragmatic clinical marker of a high-risk PTDM phenotype. This distinction has important implications. In observational transplant research, treatment exposure is often conflated with treatment effect, particularly when therapies are initiated in response to disease severity. Our findings underscore the need to interpret insulin use within the broader context of metabolic risk profiling and confounding by indication. More broadly, they suggest that PTDM should not be approached as a binary diagnostic entity but as a spectrum of metabolic phenotypes with distinct prognostic trajectories. Recognizing insulin-treated PTDM as a marker of heightened vulnerability may facilitate earlier identification of high-risk recipients, inform closer multidisciplinary follow-up, and guide stratification in future interventional trials.

From a clinical perspective, these findings carry several important implications for contemporary transplant care. First, they support the need for sustained metabolic surveillance and proactive cardiovascular risk management in recipients who develop PTDM, even when early post-transplant outcomes appear reassuring (39). The time-dependent nature of risk observed in this study suggests that metabolic injury accumulates silently and may only become clinically manifest beyond the first post-transplant years. As such, PTDM should be considered a chronic modifier of risk that warrants longitudinal attention rather than episodic intervention. Second, the identification of insulin-treated PTDM as a high-risk phenotype provides a pragmatic tool for clinical risk stratification. Insulin requirement may serve as an early marker of metabolic vulnerability, identifying recipients who could benefit from closer follow-up, intensified multidisciplinary management, and prioritization for future interventional strategies aimed at mitigating long-term graft and cardiovascular risk (40). This approach aligns with a precision-medicine framework in transplantation, in which therapeutic decisions are informed not only by graft status but also by evolving metabolic phenotypes.

More broadly, the marked heterogeneity observed across transplanted organs, eras, and geographic regions highlights the limitations of uniform PTDM management strategies. Organ-specific pathophysiology, differences in immunosuppressive exposure, and varying baseline cardiometabolic risk profiles argue for more individualized, context-specific approaches to post-transplant metabolic care (41). From a research perspective, our findings underscore the need to move beyond binary definitions of PTDM toward a more granular characterization of post-transplant metabolic phenotypes. Integration of glycemic trajectories, treatment requirements, renal function, and cardiometabolic comorbidity burden may provide a more informative framework for risk stratification and trial design than PTDM status alone (42).

This study has several strengths, including its large sample size, multinational scope, contemporary timeframe, and use of prespecified analytical strategies such as landmark analyses, propensity score matching, and organ-specific sensitivity analyses. The use of real-world electronic health record data allowed comprehensive assessment of long-term graft and patient outcomes across diverse transplant populations, capturing clinically meaningful events that are difficult to evaluate in randomized trials. Several limitations warrant consideration. As with all observational analyses, residual confounding cannot be excluded, particularly with respect to unmeasured aspects of metabolic severity, glycemic variability, adherence, or detailed immunosuppressive dosing. Furthermore, although post-transplant immunosuppressive exposures were modeled as time-dependent covariates, the TriNetX environment does not provide systematic dose-level data, precluding adjustment for glucocorticoid or calcineurin inhibitor dose intensity—a particularly relevant source of residual confounding given the marked inter-organ differences in tapering schedules and the absence of a uniform steroid-free protocol across organ types. Because exclusion of pretransplant diabetes relied primarily on diagnostic coding, occult dysglycemia may have been under-recognized in some recipients, particularly in patients with advanced organ failure such as end-stage kidney disease or advanced liver disease, where altered nutritional status and impaired insulin clearance may mask metabolic abnormalities. This limitation is inherent to large real-world electronic health record (EHR) datasets with heterogeneous baseline laboratory availability and may have biased estimates toward more conservative associations. Ascertainment of PTDM relied on EHR coding and laboratory data and may be subject to misclassification; however, the application of laboratory-based criteria and a prespecified 90-day landmark reduced misclassification and immortal time bias. Furthermore, while the 90-day landmark aligns with current consensus recommendations, the degree of immunosuppressive stabilization at this timepoint varies across organ types; some kidney transplant recipients may already be steroid-free by day 90, whereas heart and lung recipients typically remain on maintenance glucocorticoid regimens, introducing residual heterogeneity in the metabolic context at PTDM ascertainment. Laboratory trajectories were analyzed descriptively and were not used for causal inference. Finally, participating centers may disproportionately represent academic transplant programmes, potentially limiting generalizability to lower-volume settings.

In conclusion, in this large, multinational real-world cohort, PTDM emerged as a common and heterogeneous condition that modifies long-term graft and patient outcomes after solid organ transplantation. Although early post-transplant risks were comparable, PTDM was associated with a progressively higher burden of adverse outcomes over time. Among recipients with PTDM, insulin-treated individuals represent a distinct high-risk metabolic phenotype, independent of long-term glycemic control. These findings reinforce the need for refined metabolic risk stratification and sustained, phenotype-informed management strategies across the entire post-transplant continuum of care.

## Data Availability

The data that supports the findings of this study were obtained from the TriNetX Global Collaborative Network. Due to data use agreements and the federated nature of the platform, individual-level data cannot be shared or exported. Access to the data is available to participating institutions through the TriNetX Analytics platform, subject to institutional agreements and applicable regulations.
